# Handling missing data in fibromyalgia clinical trials—considerations for the use of linear mixed models in longitudinal analyses: perspectives in rheumatology

**DOI:** 10.1007/s10067-026-08193-w

**Published:** 2026-06-06

**Authors:** André Pontes-Silva, Almir Vieira Dibai-Filho, Mariana Arias Avila

**Affiliations:** 1 Postgraduate Program in Physical Education, NERD Research Group, Federal University of Maranhão, São Luís, MA Brazil; 2 Postgraduate Program in Physical Therapy, Federal University of Sao Carlos, São Carlos, SP Brazil; 3 Postgraduate Program in Physical Education, NERD Research Group — Núcleo de Estudos & Pesquisa em Reabilitação e Dor Musculoesquelética, Núcleo de Esportes, Cidade Universitária Dom Delgado, Universidade Federal do Maranhão, Avenida dos Portugueses, 1966, Vila Bacanga, CEP 65080-805 São Luís, MA Brazil

**Keywords:** Fibromyalgia, Intention-to-treat analysis, Linear mixed models, Longitudinal data analysis, Missing data, Randomized controlled trials

## Abstract

**Objective:**

To critically examine current statistical practices for handling missing data in fibromyalgia randomized controlled trials (RCTs) and to provide practical guidance for the implementation of linear mixed models (LMMs).

**Methods:**

This methodological and narrative perspective reviews recent RCTs in fibromyalgia, highlighting common limitations in the handling of missing data and longitudinal analyses. We contrast traditional approaches, such as repeated-measures ANOVA, with LMM-based strategies within the intention-to-treat (ITT) framework. Additionally, we provide a step-by-step guide for implementing LMMs in SPSS.

**Results:**

Evidence indicates that many fibromyalgia RCTs continue to rely on suboptimal statistical methods, including the exclusion of participants with incomplete data and the use of ANOVA-based approaches. These practices may reduce consistency with the ITT principle, decrease statistical efficiency, and contribute to potentially biased treatment effect estimates. In contrast, LMMs can incorporate partially observed longitudinal data, explicitly model within-subject correlations, and accommodate unbalanced longitudinal designs under the Missing At Random (MAR) assumption. However, their validity depends on correct model specification and the plausibility of underlying assumptions.

**Conclusions:**

The persistent gap between methodological recommendations and analytical practices in fibromyalgia RCTs may compromise the interpretability and reliability of treatment effect estimates. LMMs may represent a more flexible and methodologically appropriate approach for longitudinal analyses involving incomplete follow-up data, particularly when aligned with study design characteristics and available statistical expertise. Improving statistical rigor in this field remains essential to support more reliable clinical interpretation and evidence-informed decision-making.

Randomized controlled trials (RCTs) in fibromyalgia frequently face substantial dropout and incomplete longitudinal data [1]. Although the clinical determinants of dropout have been investigated [2], the statistical handling of missing data in these trials remains inconsistent, despite a systematic review of dropout from exercise interventions including more than 3000 people with fibromyalgia [3]. To address this clinical and scientific challenge, it is recommended to treat patients using the intention-to-treat principle and analyze data using linear mixed models (LMMs) [4].

The relevance of longitudinal analytical approaches such as LMMs extends beyond fibromyalgia and has been increasingly recognized across several rheumatologic and musculoskeletal conditions characterized by repeated assessments, symptom fluctuation, and incomplete follow-up data. Recent randomized trials in rheumatoid arthritis, for example, have also adopted longitudinal modeling strategies to better account for correlated repeated measures and variability in treatment response [5].

This article goes beyond reiterating well-established methodological recommendations by (i) illustrating current analytical limitations in recent fibromyalgia RCTs, (ii) contextualizing these issues within the specific challenges of this population, and (iii) providing a practical guide for implementing LMMs in widely used statistical software.

Importantly, this article is not intended to be a systematic methodological review of all fibromyalgia RCTs. Rather, it is a narrative and clinically oriented methodological perspective that uses selected recent studies as illustrative examples of persistent analytical challenges in the field. Specifically, this article addresses four interconnected gaps: (i) missing longitudinal data are highly prevalent in fibromyalgia trials, (ii) the analytical handling of missing data is often insufficiently reported or suboptimally conducted, (iii) despite longstanding methodological recommendations supporting LMMs in longitudinal research, their implementation in fibromyalgia RCTs remains inconsistent, and (iv) practical guidance tailored to clinicians and applied researchers in rheumatology remains limited [6, 7].

Although these methodological challenges are not exclusive to fibromyalgia, this condition presents particularly complex longitudinal characteristics that may exacerbate the consequences of inadequate statistical handling of missing data. Fibromyalgia is characterized by fluctuating symptoms, high variability in treatment response, and substantial dropout rates due to pain exacerbation, fatigue, and psychosocial factors. These characteristics may increase the complexity of missing data mechanisms, potentially involving deviations from the Missing At Random (MAR) assumption, and amplify the risk of biased estimates when inappropriate statistical methods are used. These challenges are particularly relevant in rheumatology, where chronic, fluctuating conditions such as fibromyalgia require longitudinal designs to capture treatment effects over time [8].

Despite the theoretical and methodological consensus regarding the advantages of LMMs, examples from recent fibromyalgia RCTs and broader methodological literature indicate that inappropriate handling of missing data and reliance on suboptimal statistical approaches remain common in clinical trials—including those conducted in fibromyalgia populations. This gap between methodological recommendations and actual analytical practices may influence treatment effect estimates and potentially contribute to less reliable clinical interpretations and decision-making in this population [8]. Accordingly, the aim of this article is to discuss the practical implications of missing data handling in fibromyalgia RCTs, illustrate common analytical limitations using recent clinical examples, and provide an accessible guide for implementing LMMs in longitudinal analyses.

Several fibromyalgia RCTs in rheumatology have used analytical approaches that may not fully exploit the flexibility of LMMs for handling incomplete longitudinal data in intention-to-treat (ITT) analyses [4]. For example, Li et al. investigated the therapeutic effects and safety of warm acupuncture therapy for fibromyalgia patients with increased cold sensitivity [9]. Although the study addresses an important clinical question, the conclusions may be limited by the analytical strategy used, as participants who discontinued treatment were not included in the analysis [4]. Additionally, the data were analyzed using ANOVA rather than LMMs [10].

A similar methodological limitation can be observed in other fibromyalgia RCTs investigating neuromodulation interventions [11]. For example, Lopes-Alves et al. conducted a randomized clinical trial evaluating home-based transcranial direct current stimulation in which 48 participants were initially randomized, but only 43 were included in the final analysis using a modified intention-to-treat approach that excluded individuals who completed less than 50% of the intervention sessions. Such analytical decisions may introduce attrition bias and compromise the validity of treatment effect estimates in longitudinal studies. Moreover, the statistical analyses primarily relied on pre–post comparisons and traditional parametric tests rather than models specifically designed for repeated-measures data. In this context, LMMs could provide a more robust analytical framework by incorporating all available observations and appropriately modeling within-subject correlations over time [11].

A comparable limitation can also be observed in the randomized trial conducted by Vicente‐Mampel et al., which investigated the effects of dry needling on pain sensitivity in individuals with fibromyalgia. In that study, although 120 participants were initially randomized, 24 discontinued participation during follow-up, resulting in a final sample of 96 participants who completed the study. Such attrition introduces important analytical challenges in longitudinal trials, particularly when treatment effects are evaluated across multiple time points. Despite repeated assessments at baseline, immediately post-intervention, and 24 h after treatment, the statistical analysis relied primarily on analysis of covariance within a traditional repeated-measures framework. In the presence of incomplete follow-up and correlated observations over time, reliance on conventional ANOVA-based strategies may produce inefficient estimates and reduce statistical power. In contrast, LMMs may provide a more flexible analytical framework, as they can incorporate partially observed longitudinal data while explicitly modeling within-subject correlations and accommodating unbalanced longitudinal data [12].

Collectively, these examples suggest that suboptimal handling of longitudinal data and missingness is not isolated but may represent a broader pattern in fibromyalgia clinical trials, highlighting a gap between methodological recommendations and analytical practices. Importantly, these observations should not be interpreted as evidence that repeated-measures ANOVA is inherently invalid. Rather, the appropriateness of each analytical strategy depends on the study design, the missing data mechanism, the covariance structure, and the research question being addressed [6].

In this context, LMMs are often more appropriate in the presence of incomplete longitudinal data than repeated-measures ANOVA for analyzing longitudinal data in RCTs [10, 13, 14], especially when the ITT principle is followed and treatment is discontinued (i.e., dropout rate) [4]. This is because LMMs account for the hierarchical structure of the data by modeling the correlation between repeated observations within the same individual using random effects [10]. While repeated-measures ANOVA relies on strict assumptions, such as sphericity, LMMs offer greater flexibility. They allow for the use of different covariance structures that better capture real-world data [13].

Traditional repeated-measures ANOVA approaches often require complete data from all participants at each time point, which may lead to the exclusion of individuals with incomplete follow-up data and reduce consistency with the ITT principle [4]. Although different methods, such as multiple imputation, can be used to address missing data in ANOVA-based analyses, one potential advantage of LMMs is their ability to incorporate partially observed longitudinal data without requiring explicit imputation under the MAR assumption [15]. The ITT principle requires that all randomized participants be included in the analysis, regardless of missing data [1]. Current CONSORT recommendations emphasize transparent reporting of missing data and adherence to the ITT principle in randomized trials [16]. As such, LMMs may provide more appropriate and statistically efficient inference in the presence of incomplete longitudinal data when the MAR assumption is considered plausible and the model is correctly specified using maximum likelihood estimation [17].

However, LMMs do not automatically guarantee a “full” ITT analysis, particularly when outcome data are completely missing after randomization. Rather, LMMs are generally considered more consistent with the ITT principle because they allow the inclusion of participants with partially observed longitudinal data without requiring listwise deletion [18].

This limitation is particularly relevant in fibromyalgia research, where missingness may plausibly be associated with pain flare-ups, fatigue severity, psychological distress, or lack of treatment response. Under these circumstances, Missing Not At Random (MNAR) mechanisms cannot be excluded, and LMMs should therefore be interpreted as methods that may provide more appropriate analyses under specific assumptions rather than as approaches that inherently “solve” missing data problems [6, 18].

Recent regulatory and methodological frameworks also emphasize the importance of clearly defining estimands and aligning statistical analyses with the clinical question of interest when handling intercurrent events and missing data in RCTs [19]. However, no statistical method can fully compensate for poor trial conduct, inadequate follow-up procedures, or systematically informative dropout mechanisms. Therefore, minimizing missing data through rigorous trial design and participant retention strategies remains essential in fibromyalgia RCTs [18].

In fibromyalgia, where treatment effects are often modest and variability is high, even small analytical biases can lead to clinically misleading or biased inferences. Overestimation or underestimation of treatment effects due to inappropriate handling of missing data may contribute to potentially biased estimates, inconsistent clinical interpretations, and uncertainty in therapeutic decision-making [20].

Although LMMs offer several advantages in ITT analyses [4], they are not a universal solution to all statistical challenges in RCTs [10, 13, 14]. Their performance hinges on the assumption that data are MAR, a premise that is often difficult to verify or justify in practice [21]. For this reason, sensitivity analyses exploring alternative missing-data assumptions, including MNAR mechanisms, are increasingly recommended in longitudinal clinical trials with substantial attrition [18, 19].

Additionally, the quality of the results hinges on the correct specification of the model, including the selection of fixed and random effects, covariance structures, and distributional assumptions [22]. Model selection in LMMs also requires careful comparison of alternative model specifications (e.g., covariance structures) using information criteria such as AIC or BIC, which adds an additional layer of complexity compared with repeated-measures ANOVA approaches. Misusing LMMs or relying on default software options without conducting appropriate diagnostics can result in biased or inefficient estimates [23].

Additionally, the distinction between repeated-measures ANOVA and LMM should not be viewed as a strict dichotomy. Some modern implementations of repeated-measures ANOVA incorporate methods such as multiple imputation or maximum likelihood approaches to address missing data. These approaches enable repeated-measures ANOVA frameworks to incorporate incomplete datasets more robustly than earlier methods. However, LMMs may offer practical advantages in that they can model within-subject correlations and account for random effects without requiring data imputation or listwise deletion. Thus, although repeated-measures ANOVA methods have evolved, LMMs continue to offer greater flexibility for many longitudinal RCT settings [24].

The continued use of suboptimal statistical approaches in fibromyalgia RCTs is not merely a methodological issue but also a matter of methodological rigor and interpretative responsibility [1, 25]. Given the burden of the disease and the vulnerability of this population, maximizing the validity and interpretability of clinical trial data should be considered an important methodological priority rather than a secondary analytical consideration [18, 19].

Finally, LMMs may help preserve statistical power and reduce the risk of bias, even when there are unbalanced designs or participant attrition, both of which are common in fibromyalgia RCTs. LMMs may facilitate potentially less biased estimation of time-by-group interactions, potentially providing a more appropriate representation of treatment effects over time [26, 27]. For these reasons, LMMs are widely recommended for longitudinal RCT data (Table 1), particularly in conditions such as fibromyalgia, where missing data, symptom fluctuation, and heterogeneous treatment responses are inherent challenges. However, the choice of model should be guided by the study design, the nature of the data, and the available statistical expertise [26, 27].

**Table 1:** General comparison between repeated-measures ANOVA and LMM in the context of RCTs

Criterion	ANOVA	LMM	General advantage of the LMM
Model flexibility	Limited	High	Greater adaptability
Missing data	Excludes subjects with any missing values	Includes subjects with partial data	Better respects ITT
ITT	May require imputation strategies	More compatible with ITT principles when partial follow-up data are available	Better accommodates incomplete longitudinal data
Correlation between measurements	Assumes sphericity (limited)	Directly models correlation	More realistic modeling
Unbalanced design	Problems with unequal group sizes	Handles unbalanced designs	Greater flexibility
Random effects (e.g., subject)	Not supported	Supported (e.g., intercepts and slopes)	Better fit for repeated measures
Loss to follow-up	Leads to exclusion	Retains subjects with available data	Preserves sample size
Error estimation	May under/overestimate	Allows separate variances	More robust estimation
Practical applicability	Less recommended without imputation	Strongly recommended for longitudinal data	Widely accepted in modern RCT analysis

## Implementing linear mixed models in SPSS: a step-by-step guide

Because many clinical researchers use SPSS, we provide a simplified step-by-step guide to implementing a LMM in this software. To run a LMM in SPSS, first open your dataset. Then, go to Analyze > Mixed Models > Linear. In the new dialog box, assign your subject ID to the Subjects field to indicate repeated measures within individuals. Then, click Continue. In the repeated tab, define the within-subject variable (e.g., time) and select an appropriate covariance structure (e.g., unstructured). Next, move the outcome of interest to the “dependent variable” cell, and move the “Group” and “Time” variables to the “factors” cell. Next, click the Fixed tab and include your fixed effects, which are usually the main effects of Group and Time, as well as the Group × Time interaction. In the random tab, add a random intercept for subject (or random slopes, if needed) to model within-subject variability. In the estimated marginal means section, select post hoc for the estimated marginal means of group, time, and group × time interaction (usually Bonferroni). Then, you may use the statistics and plots buttons to request estimates and visualize interactions. Finally, click OK to run the model or PASTE to change the syntax and add other interactions (e.g., additional interaction terms if theoretically justified). SPSS uses maximum likelihood estimation and can incorporate partially observed longitudinal data under the MAR assumption, reducing the need for listwise deletion.

Researchers should also consider whether restricted maximum likelihood (REML) or full maximum likelihood (ML) estimation is more appropriate for their objectives, particularly when comparing nested models or estimating variance components (Linear Mixed Models: A Practical Guide Using Statistical Software).
